# Supplementary material for: Modern Development and Production of a New Live Attenuated Bacterial Vaccine, SCHU S4 ΔclpB, to Prevent Tularemia
Source: Pathogens. 2021 Jun 23;10(7):795. doi: 10.3390/pathogens10070795 (PMC8308573; doi:10.3390/pathogens10070795)
Supplement: Supplementary file 1 [file pathogens-10-00795-s001.zip › pathogens-1257384-supplementary.pdf]

Supplementary Materials

Table S1. FTT designations for genes in Table 1.

FTT Number	Gene	FTT Number	Gene
FTT_0557	*ahcP*	FTT_0444	*tet*
FTT_0805	*capB*	FTT_0245	*usp*
FTT_0715	*chiX*	FTT_0023c	*0023*
FTT_1528-31	*fadAD2*	FTT_0024c	*0024*
FTT_1528	*fadD2*	FTT_0029c	*0029*
FTT_0249	*feoB*	FTT_0069c	*0069*
FTT_0733	*gpx*	FTT_0086	*0086*
FTT_0630	*hfq*	FTT_0919	*fupB*
FTT_0721c	*katG*	FTT_1023c	*1023*
FTT_0961	*mdaB*	FTT_1149c	*1149*
FTT_0125	*oppD*	FTT_1564	*1564*
FTT_0556c	*oxyR*	FTT_0918	*fupA*
FTT_0449	*pckA*	FTT_1181c	*ggt*
FTT_1354/1709	*pdpC*	FTT_1358c	*iglB*
FTT_1360c	*pdpD*	FTT_1357c	*iglC*
FTT_1209c	*pepO*	FTT_1356c	*iglD*
FTT_1699-FTT1716	*PI1*	FTT_1531c	*gplX*
FTT_1344-1361	*PI2*	FTT_1452c	*wbtC*
FTT_0980c/0981/0982	*pilAEV*	FTT_1455c	*wbtI*
FTT_1133	*pilB*	FTT_1769c	*clpB*
FTT_1134	*pilC*	FTT_1561	*kdtA*
FTT_1156c	*pilQ*	FTT_1235c	*lpcC*
FTT_0088	*pilT*	FTT_1721c	*purF*
FTT_1557c	*pmrA*	FTT_0458	*sspA*
FTT_1665	*pyrB*	FLT_0439	*0439*
FTT_0094c	*qseQ*	FTT_0085c	*0085*
FTT_0522-23	*RD5*	FTT_1275	mglA
FTT_1071	*RD8*		
FTT_1750	*recA*		
FTT_1508c	*relA*		
FTT_0178c	*rimK*		
FTT_0879	*sodC*		

Table S2. Analytical assays for master cell bank produced at Ology.

Parameter	Test Method	Specification
**Strain ID/Purity**	Whole genome sequencing	Identity confirmed
**Modified microbial limits**	Purity	Absence of contaminating organisms
**Viability**	CFU/mL	Report result
**Gram stain**	Gram stain	Gram negative coccobacillus
**Morphology**	TBD	Round, smooth and slightly mucoid single colonies
**Endotoxin**	LAL	<5 EU/kg/dose[1]
**Antibiotic Resistance**	Susceptible to tetracycline, levofloxacin, gentamycin, chloramphenicol, ciprofloxacin, streptomycin, rifampin	Negative

[1], depending on the safety margin required by the regulator, the required test dose of *ΔclpB* could fail this test. Because it possesses intact LPS albeit with low endotoxicity.

Table S3. Characterization of 25 L production run.

Test	Test/Inspection Method	Limits
Culture Morphology	Cellular morphology	Round, smooth and slightly mucoid, single colony types
	Gram stain	Gram negative coccobacilli
Serial dilution method for bacterial viability (CFU/mL)	Viable count	Plate counting 10^7 – 10^{11} CFU/mL
Endotoxin (LAL)	Endosafe Nexgen PTS	Report results[1]

[1], depending on the safety margin required by the regulator, the required test dose of *ΔclpB* could fail this test. Because it possesses intact LPS albeit with low endotoxicity.

Table S4. Selected cytokine and chemokine responses in serum 4 days after ID vaccination with ~10^5 CFU *ΔclpB*.

		Mean fold change in serum cytokine and chemokine levels 4 days following ID administration of 10^5 CFU *clpB*; n = 4 – 5 mice/group[1] (naïve serum background)						
		**IFN-γ**	**TNF-α**	**IL-6**	**RANTES**	**MCP-1**	**MIP-1β**	**KC**
Expt#								
1	Original[2]	9458 (2.22)	8.7 (6.2)	26.2 (13.8)	13.5 (16.8)	79.0 (12.8)	7.0 (10.7)	71.7 (32.2)
2	Original[2]	1850 (8.56)	4.1 (10.4)	69.6 (3.9)	3.4 (42.6)	10.0 (44.9)	5.2 (39.9)	35.1 (38.8)
3	Original[2]	3544 (5.40)	2.4 (16.3)	18.2 (14)	4.4 (17.0)	9.3 (61.8)	8.1 (25.2)	11.1 (54.3)
	New[3]	3425	7.4	48.8 (	3.9	8.2	7.2	21.2
4[3,4]	Flask	1216 (12.15)	11.6 (3.9)	18.7 (12.4)	3.4 (12.1)	29.5 (14.2)	5.4 (18.9)	22.3 (55.7)
	Ferm 27h	1895	15.0	24.3	3.5	50.9	8.2	29.1
	Ferm 29h	1578	15.3	37.7	3.0	45.3	7.5	30.7
	Ferm 42h	1132	12.1	23.2	3.4	41.9	5.4	35.4
5[3,5]	3 year 4C	3366 (5.40)	7.9 (7.1)	73.7 (3.9)	14.5 (9.1)	39.4 (15.4)	3.1 (60.6)	149 (12.6)
	3 year -20C	3125	5.3	76.3	12.1	28.0	2.9	94.1
	3 year -80C	3312	4.6	60.0	6.3	29.3	2.4	59.7
6	New[3]	13248 (1.14)	52.5 (2.8)	256 (3.7)	2.5 (44.4)	70.1 (19.9)	1.7 (12.4)	26.3 (88.0)
	CMO ferm[6]	17854	35.3	178	3.1	30.1	3.2	30.2
7	CMO ferm[6]	19704 (1.03)	12.4 (8.2)	56.9 (8.6)	3.2 (24.4)	23.1 (28.6)	3.5 (53.6)	35.3 (61.5)
	CMO lyoph[7]	14769	5.2	28.4	4.9	24.1	3.2	13.3
	New[3]	17602	5.3	41.0	4.5	18.9	3.9	14.2
	Original[3]	13051	2.8	26.4	5.2	15.3	2.6	7.7
8	CMO ferm[6]	16133 (1.03)	47.4 (1.5)	67.9 (4.2)	18.7 (8.9)	136 (6.9)	236 (1.5)	97.5 (11)
	New[3]	18552	40.1	99.3	16.1	98.9	184	100
	Original[2]	14751	26.1	11.0	10.4	68.6	143	65.1
	New x 22[3,8]	17619	20.5	50.5	11.9	74.0	77.4	43.0

[1] mean of sample/(mean of naïve mouse serum); [2]NRCC original stock *ΔclpB*; [3] new stock of *ΔclpB* prepared for vaccine development; [4] NRC fermenter run 3 (Table 5); [5] lyophilized *ΔclpB* stored at +4°C, -20°C, or -80°C, then reconstituted after 3 years; [6] 10x concentrated *ΔclpB* from Ology Biosciences fermenter run (provided frozen); [7] Ology Biosciences fermenter growth lyophilized at 10^X CFU/ml; [8] new stock *ΔclpB* passaged 22 times (daily) in MCPH broth.

Table S5. *ΔclpB* organ burdens and virulence following ID or IN challenge.

Expt	*clpB* sample	Log_{10} CFU	*clpB* in organs 4 days after ~10^5 CFU ID (n = 4 – 10)			Survival after ~10^4 CFU IN or 10^5 ID *clpB* in days (TTD) (n = 4 – 10)			
		Inoculum x 10^5	Skin	Spleen	Liver	Inoculum x 10^5	Survival	Inoculum x 10^4	% Survival
1	Original stock	2.0	5.2 ±0.2	6.7 ±0.4	5.9 ±0.4	2.0	100	NT	
2	New stock	1.5	4.9 ±0.3	6.4± 0.4	5.6± 0.5	1.5	100	NT	
3	Original stock	0.7	5.1 ±0.4	6.2± 0.2	5.9 ±0.2	0.7	100	0.7	100
	New stock	1.3	5.6 ±0.7	7.1 ±0.8	6.6 ±0.8	1.3	100	1.3	60 (6,7)
4	Flask	1.2	5.1 ±0.4	6.3 ±0.3	5.7 ±0.3	1.2	80 (4,7)	1.7	100
	Fermenter 27 h	0.8	5.6 ±0.3	6.5 ±0.3	6.0 ±0.3	0.8	100	0.3	100
	Fermenter 29 h	1.3	5.6 ±0.7	6.6 ±0.7	6.2±0.6	1.3	100	1.9	100
	Fermenter 42 h	1.4	5.1 ±0.4	6.3 ±0.3	5.7 ±0.3	1.4	100	1.8	100
5	3 year +4°C	0.6	5.3 0.9	6.5 0.6	6.0 0.4	0.6	100	0.6	100
	3 year -20°C	1.0	5.1 0.3	5.9 0.2	5.9 0.5	1.0	100	1.0	40 (7,8,11)
	3 year -80°C	0.8	4.7 0.4	6.2 0.3	6.0 0.2	0.8	100	0.8	80 (7)

6	New stock	1.6	5.9 ±0.8	6.9 ±0.9	5.6± 0.4	1.6	100	1.6	40 (7,8,8)
	Ology 10x fermenter	1.5	5.4 ±0.5	6.5 ±0.3	6.0 ±0.2	1.5	80	1.5	0 (7,7,7,7,7)
7	Ology 10x ferm	0.9	5.4 ±0.6	7.0 ±0.7	6.4 ±0.9	0.9	40 (6,6,6)	0.9	80 (5,6)
	Ology lyoph	0.6	5.1 ±0.6	6.2 ±0.3	5.6 ±0.4	0.6	100	0.6	90 (7)
	New stock	0.05	5.0 ±0.3	5.9 ±0.6	5.7 ±0.9	0.05	100	0.05	100
	Original stock	0.3	5.1 ±0.7	5.9 ±0.1	5.5 ±0.2	0.3	100	0.3	90 (28)
8	Ology 10x ferm	1.2	5.4 ±0.6	7.0 ±0.9	6.3 ±0.3	2.9	100	2.9	66.7 (6,6,6)
	New stock	1.6	5.6 ±0.4	6.6 ±0.5	6.5 ±0.4	4.1	100	4.1	44.5 (6,7,7,7,7)
	Original stock	0.9	5.5 ±0.4	6.6 ±0.3	6.1 ±0.3	4.1	100	4.1	88.9 (9)
	22x passage	1.6	5.0 ±0.2	6.2 ±0.2	5.9 ±0.2	2.1	100	2.1	88.9 (9)
